# Supplementary material for: Synthesis, Structure, and Electrochemical Properties of 2,3,4,5-Tetraphenyl-1-Monophosphaferrocene Derivatives
Source: Molecules. 2023 Mar 8;28(6):2481. doi: 10.3390/molecules28062481 (PMC10056091; doi:10.3390/molecules28062481)
Supplement: Supplementary file 1 [file molecules-28-02481-s001.zip › molecules-2222464-supplementary.pdf]

Supplementary Information for:

Synthesis, structure, and electrochemical properties of 2,3,4,5-tetraphenyl-1-monophosphaferrocene derivatives

Almaz A. Zagidullin[1], *Alena R. Lakomkina*[1,2], *Mikhail N. Khrizanforov*[1,2], *Robert R. Fayzullin*[1], *Kirill V. Kholin*[1,3], *Tatiana P. Gerasimova*[1], *Ruslan P. Shekurov*[1], *Ilya A. Bezkishko*[1], *Vasili A. Miluykov*[1]*

[1] Arbuzov Institute of Organic and Physical Chemistry, FRC Kazan Scientific Center, Russian Academy of Sciences, 8 Arbuzov Street, 420088 Kazan, Russia

[2] A.M. Butlerov Chemistry Institute of the Kazan Federal University, 18 Kremlevskaya Street, 420008 Kazan, Russia

[3] Department of Physics, Kazan National Research Technological University, 68 Karl Marx Street, 420015 Kazan, Russia

*Correspondence: miluykov@iopc.ru

Table of contents

Figure S1. $^{31}P\{^{1}H\}$ NMR spectrum of **2**. 3

Figure S2. ^{1}H NMR spectrum of **2**. 4

Figure S3. $^{13}C\{^{1}H\}$ NMR spectrum of **2**. 5

Figure S4. $^{31}P\{^{1}H\}$ NMR spectrum of **3**. 6

Figure S5. ^{1}H NMR spectrum of **3**. 7

Figure S6. $^{13}C\{^{1}H\}$ NMR spectrum of **3**. 8

Figure S7. Semi-derivative of CV for oxidation of **2**. 9

Figure S8. Semi-derivative of CV for oxidation of **3**. 10

Table S1. Cartesian coordinates of the optimized S_0 ground state structure of **2**. 11

Table S2. Cartesian coordinates of the optimized S_0 ground state structure of cationic form of **2** with s=1/2. 12

Table S3. Cartesian coordinates of the optimized S_0 ground state structure of cationic form of **2** with s=5/2. 13

Table S4. Cartesian coordinates of the optimized S_0 ground state structure of **3**. 14

Table S5. Cartesian coordinates of the optimized S_0 ground state structure of cationic form of **3** with s=1/2. 15

Table S6. Cartesian coordinates of the optimized S_0 ground state structure of cationic form of **3** with s=5/2. 16

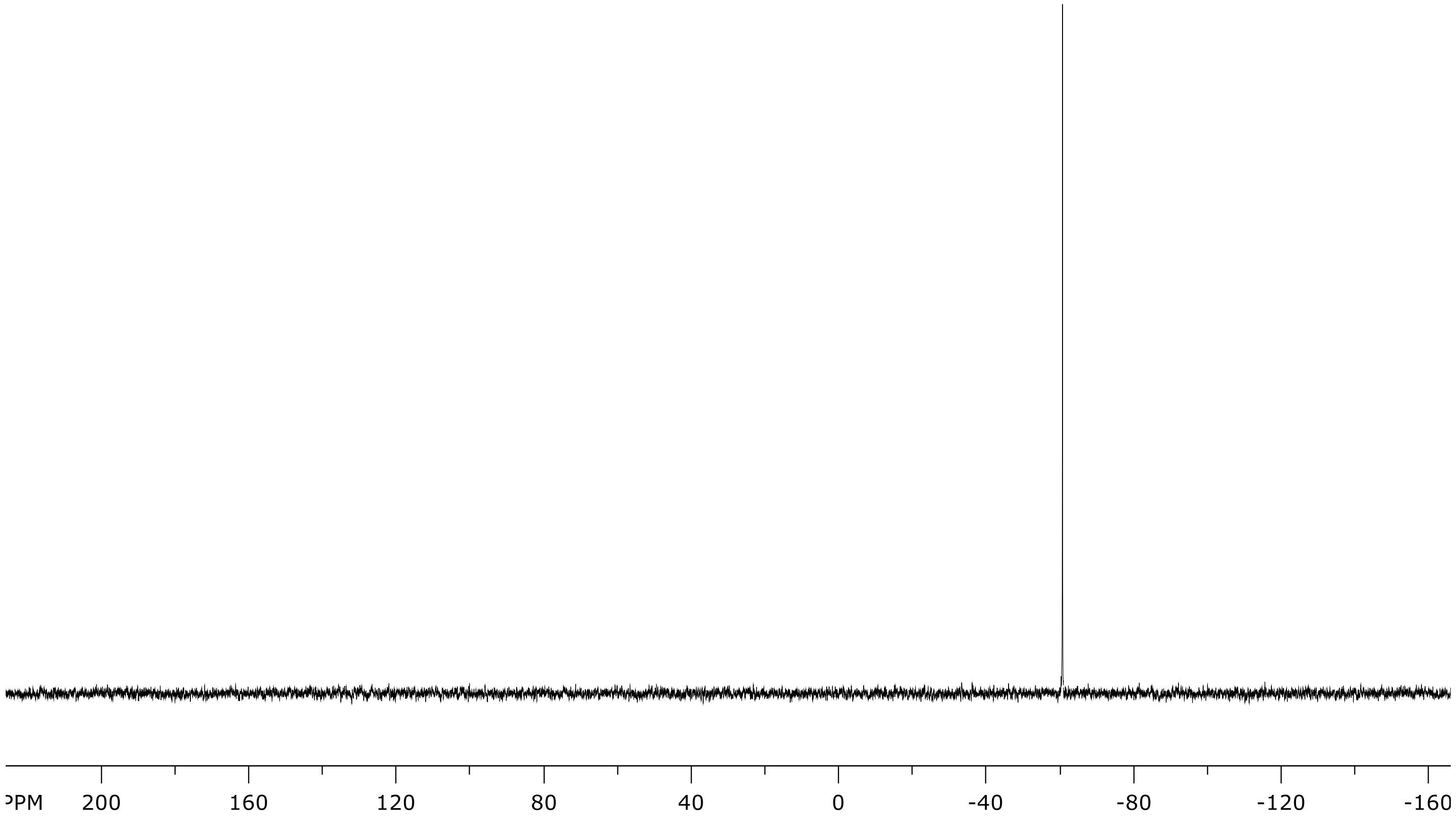

Figure S1. $^{31}P\{^{1}H\}$ NMR spectrum of **2**.

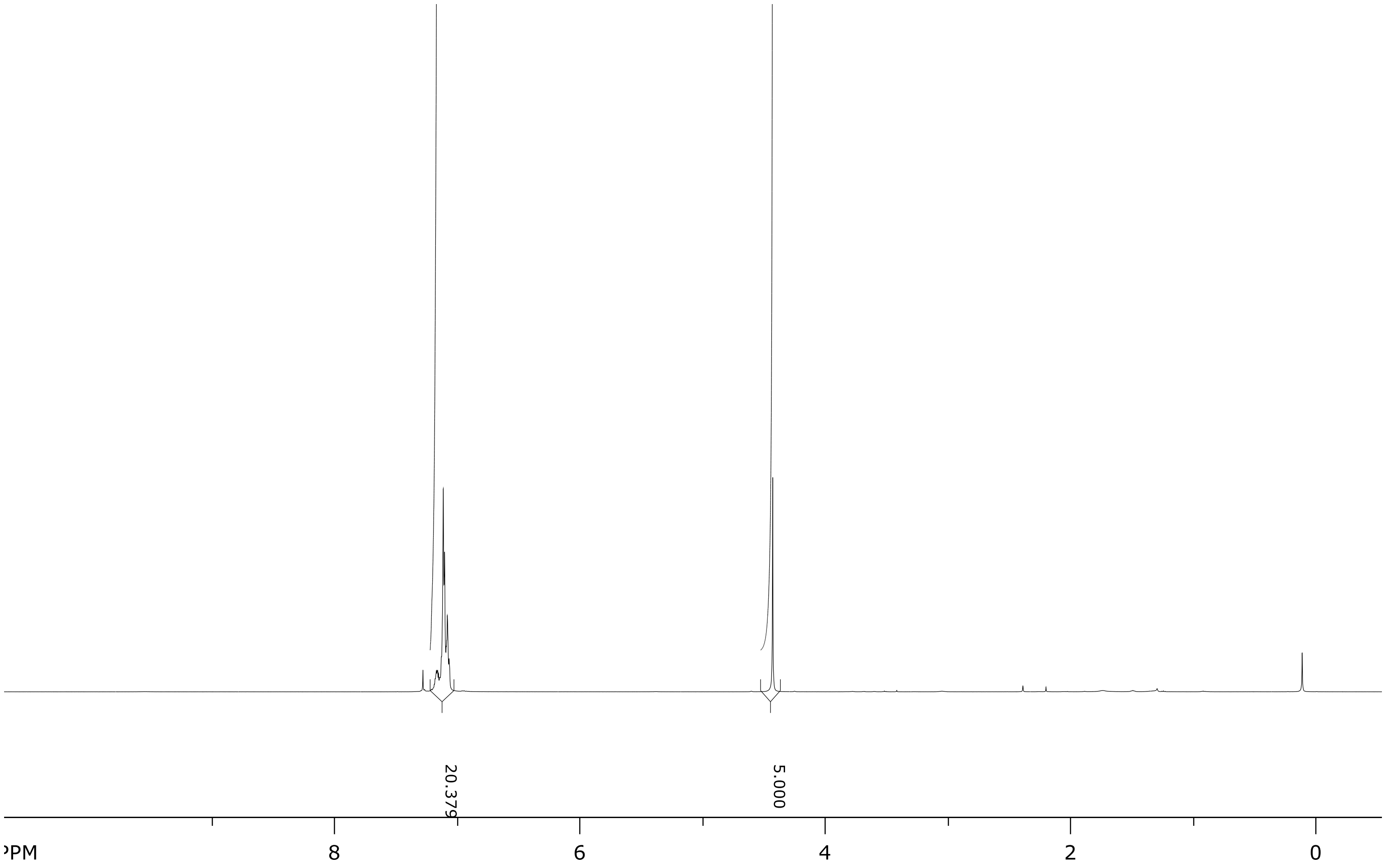

Figure S2. ^{1}H NMR spectrum of **2**.

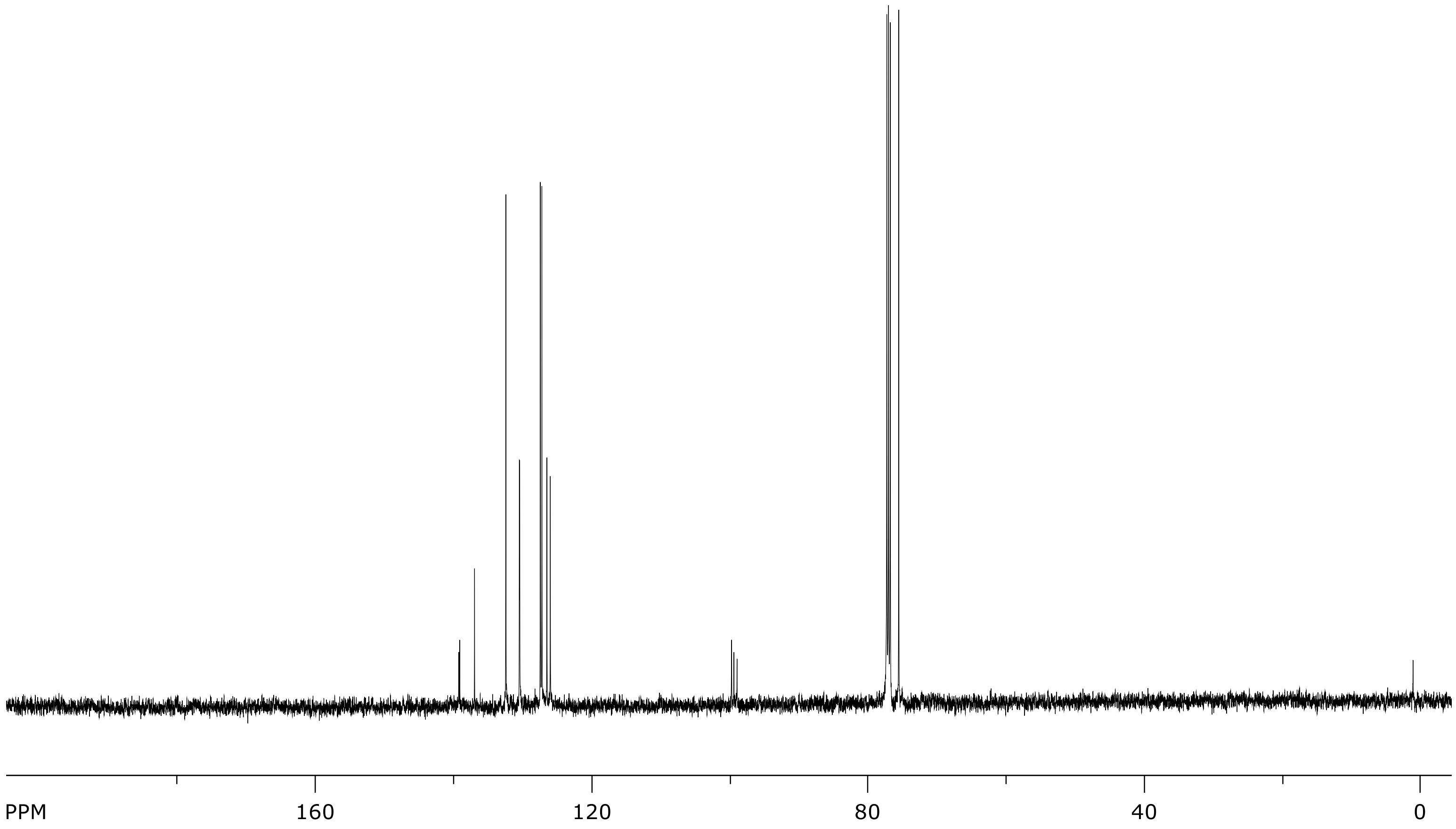

Figure S3. $^{13}C\{^{1}H\}$ NMR spectrum of **2**.

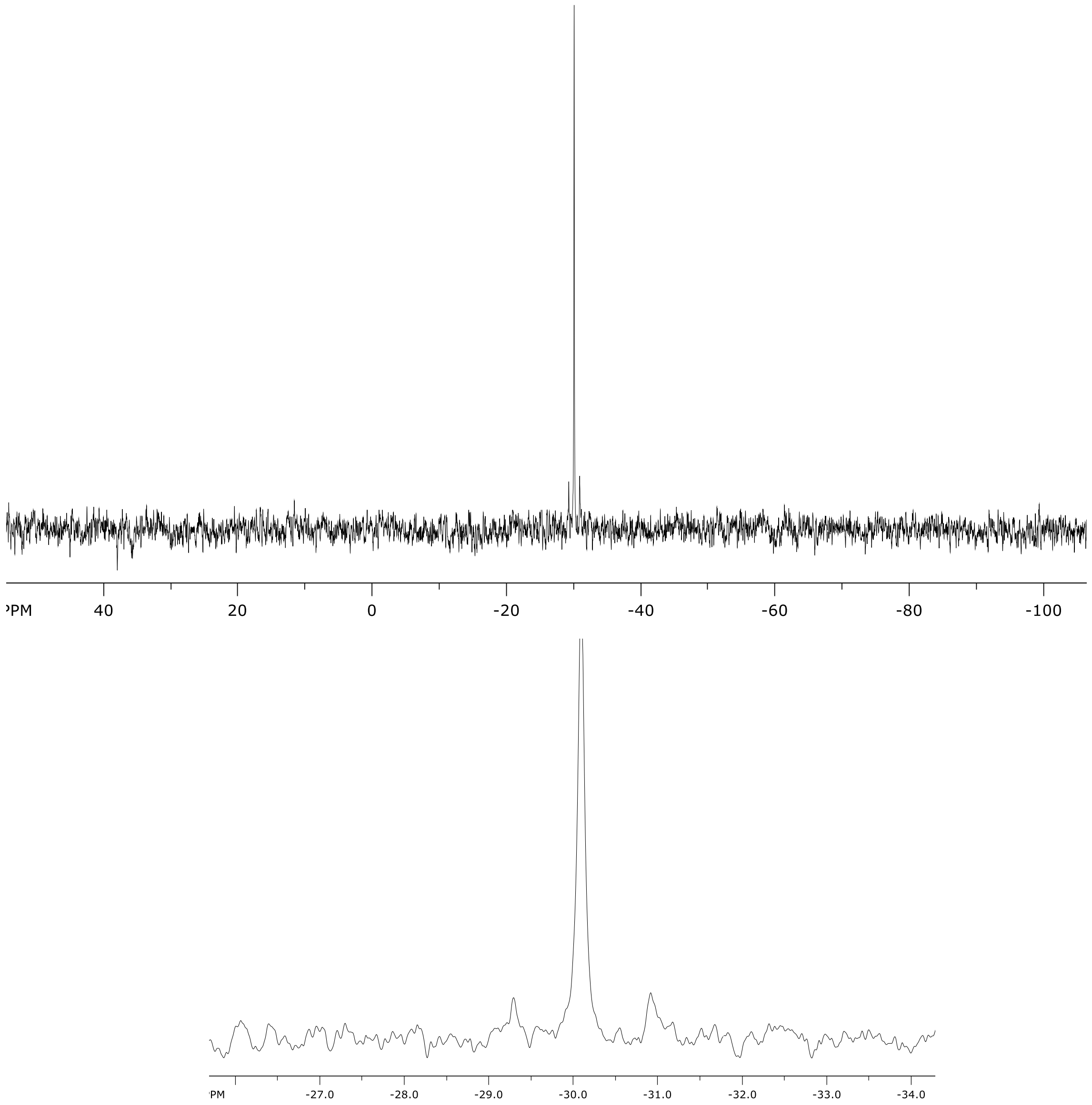

Figure S4. $^{31}P\{^1H\}$ NMR spectrum of **3**.

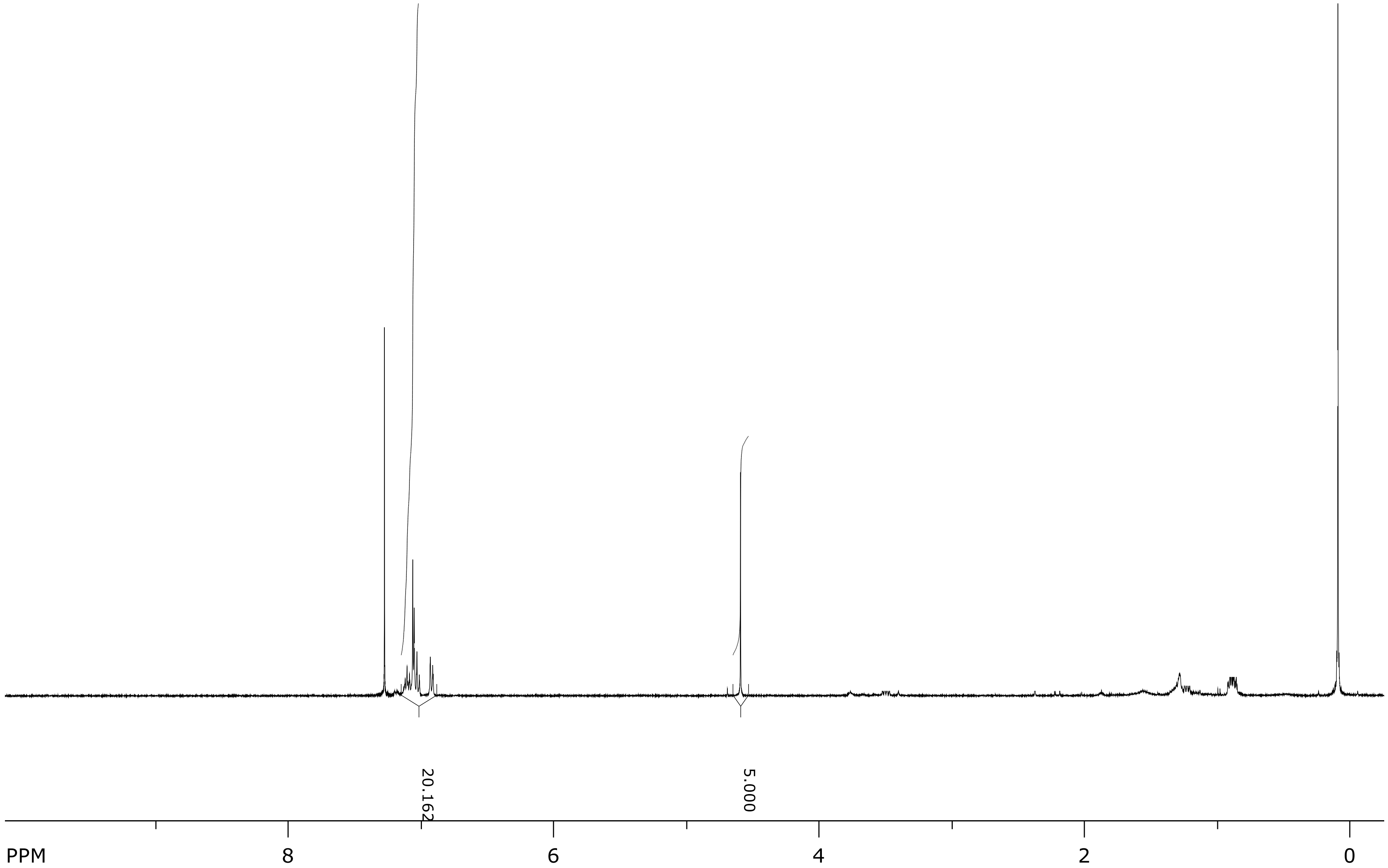

Figure S5. ^{1}H NMR spectrum of **3**.

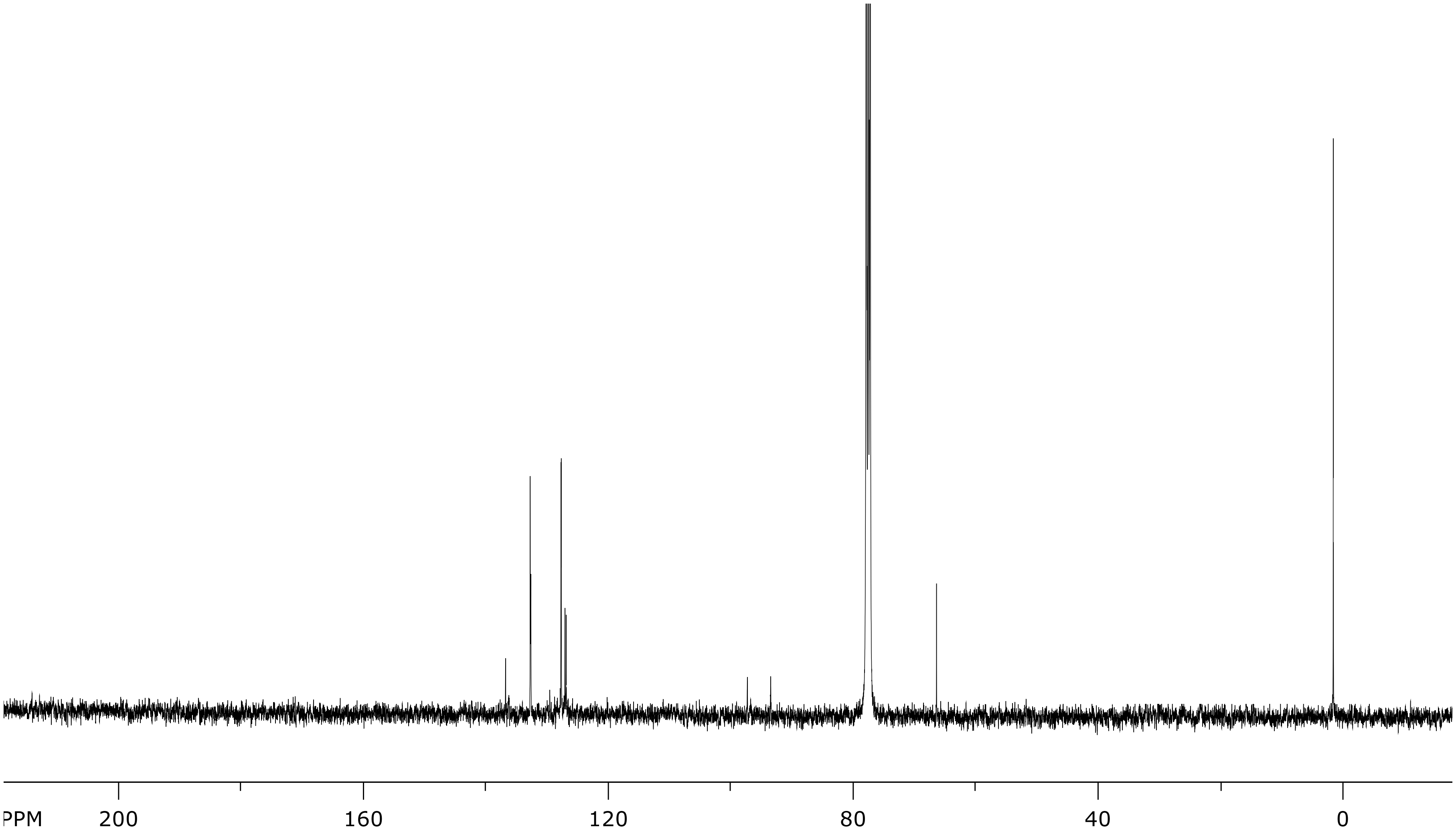

Figure S6. $^{13}C\{^{1}H\}$ NMR spectrum of **3**.

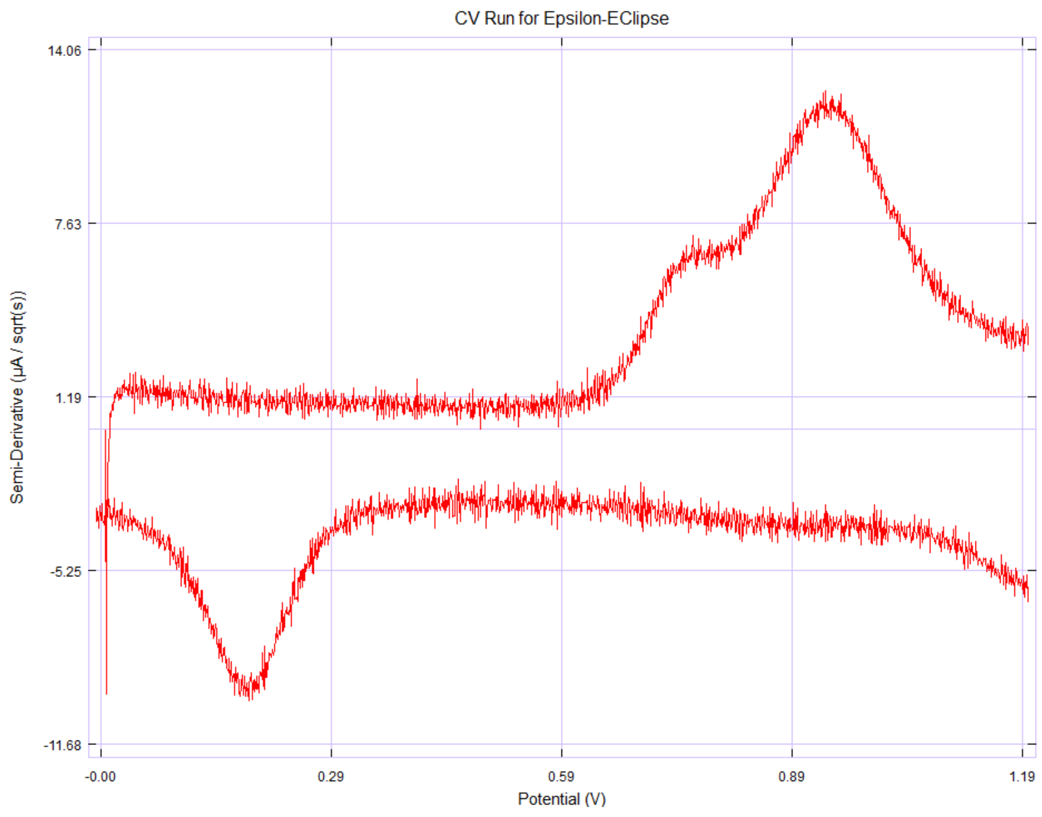

Figure S7. Semi-derivative of CV for oxidation of **2**.

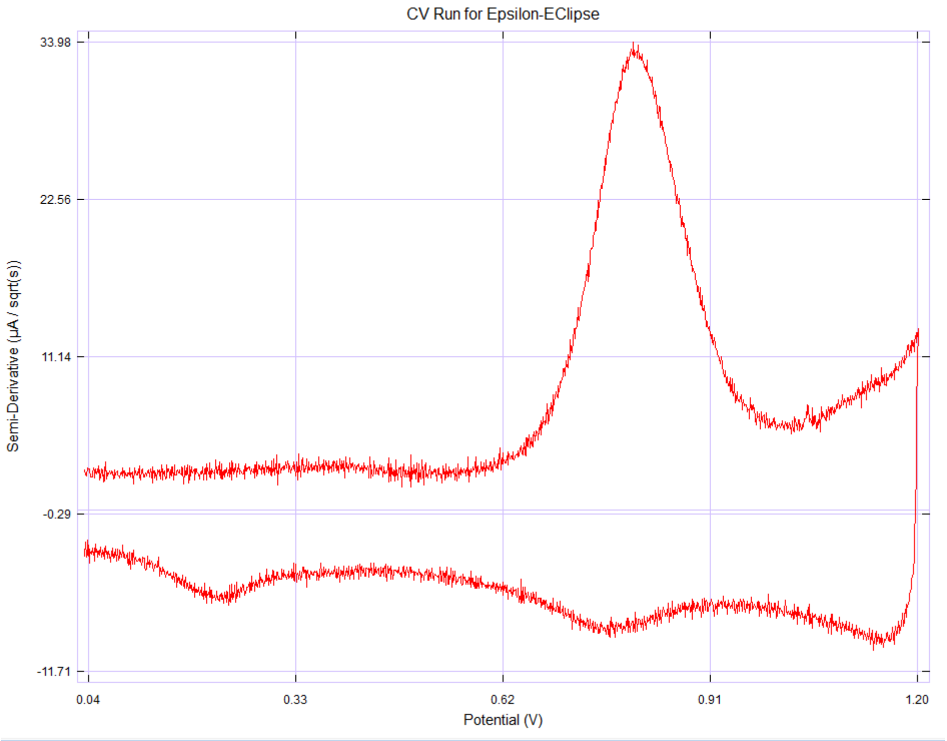

Figure S8. Semi-derivative of CV for oxidation of **3**.

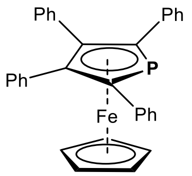

2

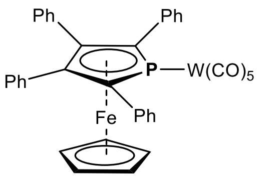

3

Table S1. Cartesian coordinates of the optimized S_0 ground state structure of **2** (E=-2876.2633135).

	X	Y	Z
Fe	-0.002583	-0.918024	0.918009
P	0.002715	-2.104648	-1.053163
C	1.259150	-0.868091	-0.732604
C	-0.719740	0.418750	-0.475319
C	-1.538391	1.634463	-0.278291
C	0.715644	0.421959	-0.470906
C	1.318363	2.764511	-1.045595
H	0.518745	2.756825	-1.777523
C	2.692429	-1.197645	-0.830470
C	1.537882	1.633541	-0.260052
C	-1.358771	2.474999	0.818609
H	-0.608386	2.220589	1.557111
C	1.176664	-1.371090	2.540616
H	2.249859	-1.480798	2.493723
C	-1.073945	-1.828284	2.410967
H	-2.008386	-2.340063	2.236016
C	0.224013	-2.401723	2.320825
H	0.443690	-3.430700	2.080768
C	3.212100	-2.324145	-0.190353
H	2.551621	-2.931004	0.419421
C	-2.118213	3.626012	0.958737
H	-1.964867	4.269882	1.817887
C	-2.694307	-1.214982	-0.763990
C	-1.257773	-0.873200	-0.745655
C	0.468714	-0.161568	2.769858
H	0.911038	0.808762	2.939670
C	-2.493587	1.974824	-1.235301
H	-2.641744	1.324207	-2.089787
C	-4.925071	-1.068042	0.162935
H	-5.595294	-0.682107	0.923424
C	2.572882	1.657336	0.673457
H	2.761750	0.773860	1.271269
C	-0.921792	-0.445524	2.692412
H	-1.719158	0.275590	2.791771
C	2.107387	3.894872	-0.892922
H	1.920533	4.766764	-1.510166
C	-3.581597	-0.735196	0.202962
H	-3.207586	-0.091050	0.988534
C	-3.070338	3.954864	0.002360
H	-3.665869	4.854366	0.112627
C	3.361975	2.786257	0.826420
H	4.163599	2.786649	1.556909
C	4.549733	-2.664093	-0.322891
H	4.934002	-3.542719	0.183967
C	-3.197444	-2.045713	-1.766202
H	-2.523699	-2.422947	-2.528384
C	-4.542892	-2.384393	-1.804099
H	-4.910979	-3.031455	-2.592822
C	3.130061	3.911188	0.045301
H	3.746266	4.795360	0.165551
C	3.549156	-0.425553	-1.617518
H	3.159395	0.446976	-2.128661
C	5.395260	-1.880686	-1.096911
H	6.442765	-2.142196	-1.197579
C	-3.253983	3.126051	-1.096039
H	-3.994408	3.374979	-1.848283
C	-5.412987	-1.896515	-0.840309
H	-6.464604	-2.159327	-0.869108
C	4.887761	-0.762321	-1.744966
H	5.538074	-0.147291	-2.357506

Table S2. Cartesian coordinates of the optimized S_0 ground state structure of cationic form of **2** with s=1/2 (E=-2876.0144827).

	X	Y	Z
Fe	-0.006130	-0.901588	0.918597
P	-0.001882	-2.091899	-1.045432
C	1.269321	-0.852590	-0.743866
C	-0.711075	0.453838	-0.515741
C	-1.525458	1.667382	-0.301972
C	0.730702	0.446570	-0.506731
C	1.324395	2.792428	-1.039121
H	0.535888	2.794340	-1.782731
C	2.698578	-1.198456	-0.829062
C	1.546768	1.654369	-0.264984
C	-1.339548	2.498014	0.801488
H	-0.579466	2.252696	1.534054
C	1.154628	-1.437266	2.557093
H	2.226900	-1.554793	2.502485
C	-1.102884	-1.862524	2.390152
H	-2.042854	-2.352584	2.183716
C	0.190957	-2.449912	2.286943
H	0.400784	-3.473120	2.015810
C	3.191536	-2.359149	-0.230082
H	2.523806	-2.990901	0.346728
C	-2.107216	3.641025	0.953554
H	-1.957811	4.279863	1.816349
C	-2.694148	-1.199266	-0.767169
C	-1.263671	-0.841113	-0.755522
C	0.458916	-0.230212	2.835185
H	0.910799	0.729128	3.038720
C	-2.483767	2.003168	-1.257361
H	-2.634422	1.360097	-2.116983
C	-4.934926	-1.037248	0.119278
H	-5.626040	-0.620261	0.842849
C	2.568574	1.663222	0.683301
H	2.765508	0.770800	1.265888
C	-0.934759	-0.494065	2.732439
H	-1.723418	0.235516	2.839709
C	2.110825	3.920516	-0.862193
H	1.933752	4.798398	-1.472584
C	-3.600926	-0.671529	0.156747
H	-3.260574	0.036565	0.902226
C	-3.059291	3.971853	-0.002646
H	-3.656989	4.868404	0.114603
C	3.344618	2.795451	0.866812
H	4.134885	2.793093	1.608663
C	4.524850	-2.710313	-0.365531
H	4.895659	-3.612174	0.107817
C	-3.159618	-2.103676	-1.723252
H	-2.472849	-2.515887	-2.455346
C	-4.498446	-2.464114	-1.762640
H	-4.844532	-3.160789	-2.517222
C	3.116579	3.927345	0.094066
H	3.726157	4.812422	0.234940
C	3.564645	-0.404712	-1.583066
H	3.193005	0.490337	-2.067134
C	5.382001	-1.909535	-1.108866
H	6.425053	-2.183571	-1.216251
C	-3.241451	3.154599	-1.109801
H	-3.979546	3.411372	-1.860738
C	-5.388774	-1.934121	-0.840927
H	-6.434595	-2.217153	-0.869390
C	4.896471	-0.761999	-1.720815
H	5.558095	-0.139926	-2.312477

Table S3. Cartesian coordinates of the optimized S_0 ground state structure of cationic form of **2** with s=5/2 (E=-2875.9955656).

	X	Y	Z
Fe	-0.248002	-1.462019	0.907055
P	-0.002053	-1.922430	-1.409983
C	1.267882	-0.753350	-0.906559
C	-0.695886	0.568300	-0.638568
C	-1.491657	1.775119	-0.350679
C	0.741759	0.524658	-0.590601
C	1.360387	2.913950	-0.888779
H	0.567166	3.009702	-1.620597
C	2.689394	-1.115860	-0.988169
C	1.575115	1.687992	-0.253828
C	-1.302498	2.508953	0.820893
H	-0.543361	2.201496	1.529653
C	1.212382	-1.422585	2.776515
H	2.284097	-1.510543	2.671626
C	-1.015217	-1.959098	2.987706
H	-1.930244	-2.522173	3.103377
C	0.295418	-2.502773	2.882253
H	0.546887	-3.553989	2.890591
C	3.145439	-2.313253	-0.430766
H	2.449288	-2.960162	0.095120
C	-2.068932	3.634254	1.076180
H	-1.915976	4.193925	1.991789
C	-2.682620	-1.047242	-0.976414
C	-1.264845	-0.671510	-1.004163
C	0.475102	-0.217523	2.824300
H	0.890310	0.777085	2.758635
C	-2.453897	2.198352	-1.268838
H	-2.605193	1.636420	-2.183732
C	-4.868546	-0.977362	0.059165
H	-5.526853	-0.593671	0.830014
C	2.611176	1.580461	0.677860
H	2.801879	0.627879	1.157649
C	-0.900145	-0.543868	2.952755
H	-1.716074	0.161430	3.016327
C	2.165049	4.003471	-0.597330
H	1.995831	4.945740	-1.105371
C	-3.551428	-0.558164	0.011801
H	-3.187851	0.157768	0.739339
C	-3.023440	4.049454	0.156411
H	-3.618615	4.933714	0.353149
C	3.399911	2.676609	0.982000
H	4.195014	2.582105	1.712611
C	4.478258	-2.677903	-0.532616
H	4.819512	-3.605601	-0.087912
C	-3.176889	-1.970865	-1.906988
H	-2.522290	-2.353939	-2.682628
C	-4.499785	-2.374550	-1.864416
H	-4.871559	-3.075941	-2.602183
C	3.179673	3.890976	0.343722
H	3.801968	4.747371	0.576654
C	3.596029	-0.300349	-1.671325
H	3.254750	0.622906	-2.123573
C	5.371669	-1.857675	-1.208413
H	6.413749	-2.143235	-1.293114
C	-3.209572	3.332741	-1.018362
H	-3.947451	3.657444	-1.742835
C	-5.348764	-1.881732	-0.880822
H	-6.383673	-2.201988	-0.846911
C	4.924728	-0.674189	-1.782155
H	5.616039	-0.037452	-2.321995

Table S4. Cartesian coordinates of the optimized S_0 ground state structure of **3** (E=-3509.7437236).

	X	Y	Z
W	2.998060	0.041178	-0.235872
Fe	-1.162350	-0.010706	1.291768
P	0.487340	0.017489	-0.273467
O	3.129532	-1.625115	2.491406
O	2.851013	2.755984	1.465282
O	3.145003	1.644920	-3.007618
O	6.161457	0.125135	-0.191963
C	-0.346364	-2.697789	-0.334925
C	3.059858	-1.026710	1.518073
C	2.904386	1.799904	0.844255
C	-0.460579	2.712653	-0.289275
C	5.016207	0.092332	-0.209343
C	-0.754501	-3.509247	-1.394267
H	-1.399272	-3.097513	-2.162224
C	-2.066809	0.692927	-0.421222
C	-0.165789	-0.250121	3.076801
H	0.884933	-0.472822	3.170914
C	-2.142131	0.898182	2.851650
H	-2.861933	1.694300	2.734375
C	-2.042801	-0.743758	-0.420629
C	-4.420039	1.259198	0.242446
H	-4.378666	0.477224	0.990424
C	-0.339613	-4.829260	-1.468129
H	-0.664112	-5.447067	-2.298129
C	3.063213	1.079437	-2.017776
C	-0.767063	1.269400	-0.320958
C	0.486223	-3.239710	0.643237
H	0.799640	-2.616100	1.470939
C	-5.219905	-2.206697	-1.813729
H	-5.892546	-2.028253	-2.645342
C	-0.727110	-1.276056	-0.303282
C	0.494267	-5.358301	-0.491420
H	0.822779	-6.389514	-0.555472
C	-2.437246	-0.491475	2.832919
H	-3.413038	-0.933836	2.699138
C	-5.577322	2.002494	0.075200
H	-6.438600	1.804234	0.703629
C	-5.634863	2.996380	-0.893344
H	-6.539630	3.579817	-1.022807
C	-1.215820	-1.200572	2.972110
H	-1.099124	-2.273612	2.957555
C	0.906542	-4.558659	0.565249
H	1.559460	-4.959967	1.332496
C	0.434347	3.242254	-1.217878
H	0.878339	2.588695	-1.958267
C	-4.592547	-3.472177	0.127290
H	-4.771335	-4.289470	0.817262
C	-1.051450	3.574593	0.634326
H	-1.761925	3.176138	1.346798
C	-4.115725	-1.386212	-1.636335
H	-3.930001	-0.569111	-2.324020
C	-3.301660	1.496026	-0.555267
C	-5.463889	-3.249943	-0.931251
H	-6.328255	-3.889922	-1.069338
C	0.161878	5.439269	-0.282783
H	0.407576	6.495219	-0.274006
C	-3.369787	2.493079	-1.527468
H	-2.505670	2.686599	-2.152513
C	0.746318	4.593912	-1.214095
H	1.449332	4.983568	-1.941926
C	-0.737600	1.047769	2.997817
H	-0.191706	1.979423	2.997666
C	-3.241333	-1.596608	-0.571229
C	-4.527782	3.237804	-1.694763
H	-4.563143	4.010838	-2.454359
C	-0.744334	4.925151	0.636424
H	-1.211402	5.580295	1.363534
C	-3.489371	-2.652708	0.303996
H	-2.801518	-2.831564	1.121461
C	3.034284	-1.737453	-1.293572
O	3.072976	-2.713995	-1.880900

Table S5. Cartesian coordinates of the optimized S_0 ground state structure of cationic form of **3** with s=1/2 (E=-3509.4856078).

	X	Y	Z
W	-2.964590	-0.058400	-0.246081
Fe	1.196310	0.009336	1.312289
P	-0.519705	-0.032789	-0.264457
O	-3.133205	1.508150	2.537358
O	-2.919982	-2.846278	1.341557
O	-3.078772	-1.567603	-3.079577
O	-6.146095	-0.119384	-0.286926
C	0.298361	2.696358	-0.296172
C	-3.067616	0.945958	1.544799
C	-2.937022	-1.865882	0.762261
C	0.482347	-2.714844	-0.265106
C	-5.008800	-0.095822	-0.274869
C	0.694051	3.511399	-1.357630
H	1.338147	3.112339	-2.132501
C	2.060624	-0.673286	-0.457095
C	0.142485	0.215915	3.097632
H	-0.913582	0.421561	3.165864
C	2.149897	-0.896626	2.928661
H	2.884503	-1.680044	2.816885
C	2.015923	0.769228	-0.449561
C	4.425711	-1.201055	0.194097
H	4.394674	-0.403209	0.926526
C	0.258392	4.824705	-1.425590
H	0.565700	5.448001	-2.257285
C	-3.015134	-1.035858	-2.074839
C	0.769876	-1.269656	-0.318627
C	-0.536026	3.220475	0.690145
H	-0.848911	2.593389	1.515778
C	5.167023	2.251786	-1.853310
H	5.833582	2.082994	-2.691051
C	0.693523	1.278988	-0.289987
C	-0.570768	5.340533	-0.438210
H	-0.910261	6.368065	-0.496273
C	2.423256	0.496243	2.934943
H	3.394664	0.955731	2.830128
C	5.583819	-1.941437	0.026970
H	6.450843	-1.731549	0.642662
C	5.636213	-2.944754	-0.932652
H	6.543945	-3.521995	-1.065316
C	1.183723	1.184553	3.029215
H	1.050745	2.255847	3.010311
C	-0.965978	4.536134	0.621739
H	-1.614460	4.931237	1.395261
C	-0.445854	-3.249715	-1.157360
H	-0.929146	-2.603827	-1.879056
C	4.545665	3.510552	0.098270
H	4.726520	4.327680	0.787047
C	1.128427	-3.568052	0.630038
H	1.874387	-3.168727	1.305457
C	4.074030	1.419010	-1.668161
H	3.892700	0.601521	-2.356244
C	3.302244	-1.462897	-0.589276
C	5.408732	3.293576	-0.968617
H	6.267338	3.939366	-1.111955
C	-0.099187	-5.443221	-0.241335
H	-0.328220	-6.502389	-0.226730
C	3.360466	-2.472561	-1.549225
H	2.492716	-2.683255	-2.163336
C	-0.732573	-4.606448	-1.147691
H	-1.453904	-5.007318	-1.850289
C	0.740183	-1.071843	3.022778
H	0.213265	-2.014186	2.992317
C	3.205936	1.630806	-0.597913
C	4.525128	-3.204324	-1.722673
H	4.562128	-3.982380	-2.476215
C	0.836910	-4.921043	0.643463
H	1.342535	-5.573160	1.346299
C	3.446452	2.688097	0.278496
H	2.759794	2.872082	1.096434
C	-3.002257	1.765917	-1.239244
O	-3.045396	2.761046	-1.787109

Table S6. Cartesian coordinates of the optimized S_0 ground state structure of cationic form of **3** with s=5/2 (E=-3509.4739125).

	X	Y	Z
W	-2.863096	0.024713	-0.194648
Fe	0.773285	0.233007	1.456433
P	-0.472718	0.004969	-0.753659
O	-2.609861	1.902204	2.395136
O	-2.479875	-2.698745	1.470875
O	-3.608935	-1.783745	-2.759197
O	-5.974052	-0.023395	0.565190
C	0.441893	2.710285	-0.626979
C	-2.650113	1.228934	1.478431
C	-2.613434	-1.743577	0.871937
C	0.352394	-2.708598	-0.659788
C	-4.873108	-0.001788	0.291006
C	0.727597	3.380409	-1.816025
H	1.224296	2.851332	-2.621777
C	2.034966	-0.756035	-0.608202
C	0.012450	-0.036207	3.519011
H	-1.022796	0.120993	3.780707
C	1.993511	-1.048506	2.949295
H	2.717714	-1.787077	2.638802
C	2.053914	0.692455	-0.539865
C	4.287818	-1.339804	0.305533
H	4.198839	-0.525058	1.013885
C	0.381006	4.713757	-1.964562
H	0.605865	5.226659	-2.892576
C	-3.320264	-1.147361	-1.866193
C	0.747191	-1.295293	-0.677721
C	-0.190236	3.399041	0.405445
H	-0.399337	2.892809	1.343027
C	5.400653	2.054860	-1.574053
H	6.159834	1.859776	-2.322540
C	0.769604	1.274879	-0.499702
C	-0.254237	5.391980	-0.931668
H	-0.526032	6.434068	-1.053011
C	2.263913	0.312856	3.197990
H	3.227867	0.794863	3.122808
C	5.425096	-2.130304	0.302852
H	6.216395	-1.936765	1.017940
C	5.554463	-3.161741	-0.618267
H	6.447092	-3.776661	-0.623596
C	1.040462	0.946716	3.546031
H	0.916165	1.986545	3.813561
C	-0.536226	4.734111	0.256940
H	-1.024298	5.259318	1.069932
C	-0.565754	-3.185612	-1.597336
H	-0.922323	-2.525428	-2.380272
C	4.585086	3.347851	0.282339
H	4.708621	4.160344	0.989008
C	0.844944	-3.586584	0.308329
H	1.568742	-3.230996	1.031230
C	4.269138	1.257640	-1.522006
H	4.148662	0.441746	-2.224650
C	3.255579	-1.580041	-0.604437
C	5.564902	3.096088	-0.669765
H	6.455321	3.712994	-0.708214
C	-0.506803	-5.364396	-0.586386
H	-0.840970	-6.394856	-0.554579
C	3.395696	-2.618765	-1.527358
H	2.605964	-2.809360	-2.244588
C	-0.990987	-4.504999	-1.562298
H	-1.697698	-4.862068	-2.302698
C	0.605419	-1.268292	3.139636
H	0.088837	-2.209325	3.016823
C	3.277202	1.506618	-0.570740
C	4.540138	-3.398830	-1.536973
H	4.640536	-4.195840	-2.264633
C	0.416418	-4.902211	0.344191
H	0.803080	-5.572380	1.103414
C	3.444077	2.565214	0.325052
H	2.675721	2.773065	1.061792
C	-3.068920	1.811037	-1.258157
O	-3.179064	2.788071	-1.822184
